# Selective Progesterone Receptor Modulators for the Medical Treatment of Uterine Fibroids with a Focus on Ulipristal Acetate

**DOI:** 10.1155/2018/1374821

**Published:** 2018-06-24

**Authors:** Thomas Rabe, Nicole Saenger, Andreas D. Ebert, Thomas Roemer, Hans-Rudolf Tinneberg, Rudy Leon De Wilde, Markus Wallwiener

**Affiliations:** ^1^Deutsche Gesellschaft für Gynäkologische Endokrinologie und Fortpflanzungsmedizin, Germany; ^2^Klinikum für Frauenheilkunde und Geburtshilfe, Frankfurt/M, Germany; ^3^Praxis für Frauengesundheit, Gynäkologie und Geburtshilfe-Endometriosezentrum Berlin, Germany; ^4^Evangelisches Krankenhaus Weyertal gGmbH, Köln, Germany; ^5^Universitätsklinik Gießen und Marburg, Campus Gießen, Germany; ^6^Klinik für Frauenheilkunde, Geburtshilfe und Gynäkologische Onkologie; Universitätsklinik für Gynäkologie, Pius-Hospital Oldenburg, Germany; ^7^Universitäts-Frauenklinik Heidelberg, Germany

## Abstract

Uterine fibroids are the most frequent benign tumours in women of child-bearing age. Their symptoms are diverse and the quality of life of the women affected can be significantly impaired. While treatment to date has been primarily by means of surgical intervention, selective progesterone receptor modulators (SPRMs) open up new medication-based treatment options. EMA's Pharmacovigilance Risk Assessment Committee (PRAC) has recently completed its review of ESMYA® (ulipristal acetate, 5 mg), following reports of serious liver injury, including liver failure leading to transplantation in postmarketing settings. We will provide some information on the PRAC's recommendations to minimize this risk. Nevertheless, the effectiveness and safety of the SPRM ulipristal acetate (UPA), both with regard to preoperative administration and with regard to an intermittent administration as long-term treatment for patients with symptomatic uterine fibroids, have been shown in several clinical studies (PEARL I–IV).

## 1. Introduction

Uterine fibroids (synonym, leiomyomata) are benign tumours originating from the smooth musculature of the uterus, the myometrium. They consist of smooth muscle cells and fibroblasts, which are embedded in the abundant extracellular matrix and can reach a considerable size. They grow intramurally, in the subserous or in the submucous layer, and are classified into 8 different types [1].

### 1.1. Clinical Symptoms

Fibroids occur in women of child-bearing age and may lead to a significant limitation of their quality of life [2]. The clinical symptoms include menstrual disorders (e.g., hypermenorrhea, menorrhagia, and dysmenorrhoea), right up to anaemia. The increase in the size of the uterus can lead to gastrointestinal symptoms (e.g., obstipation), voiding disorders (e.g., residual urine, nocturia, and pollakisuria), and pain or a sensation of pressure in the pelvic or abdominal region. Further possible manifestations are fertility disorders or recurrent miscarriages. Patients with fibroids, however, may also have no medical complaints at all [3, 4].

### 1.2. Risk Factors

Age and ethnicity are the most important risk factors for the occurrence of fibroids. No fibroids have been described in prepubescent girls to date. Fibroids first develop during adolescence, with increasing incidence until menopause. In the USA, an incidence of uterine fibroids in women of African ancestry, increased by two to three times in comparison with Caucasian women, has been observed [5]. Nulliparity, early menarche, a history of dysmenorrhoea, a family history of fibroids, genetic factors, and a high body mass index (BMI) have been mentioned as further possible risk factors. The risk can also be increased by hypertension and diabetes [4–6].

### 1.3. Epidemiology

**Figure 1:**
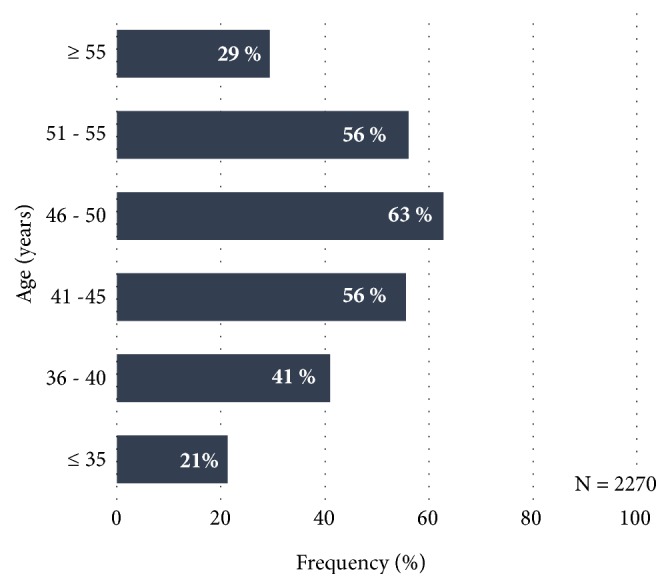
Prevalence of fibroids according to age-group, as percentages (N = 2,270), according to Ahrendt et al. [7].

Fibroids are the most frequent benign, solid tumours of the female genital tract [7]. In a prospective study of the prevalence of fibroids in Germany, Ahrendt et al. describe the data of 2,296 women who visited out-patient gynaecology facilities and were not less than 30 years of age [7]. Fibroids were detected in 41.6% of the women by means of transvaginal sonography. Age-dependence was able to be demonstrated. With increasing age, the prevalence of the fibroids in premenopause increased from 21.3% (30 – 35 years of age) to 62.8% (46 – 50 years of age). The prevalence declined again in patients over 50 years of age. In patients over 55 years of age, these reached a value of 29.4% (Figure 1).

In this study, the occurrence of fibroids did not correlate either with the age at menarche or with the body mass index. Due to their results, the authors assume that more than 40% of the women over 30 years of age suffer from fibroids and that more than 50% of women in Germany develop uterine fibroids at some time in their lives.

### 1.4. Pathogenesis

To date, the exact pathogenesis of uterine fibroids is largely unexplained. Thus far, aside from sexual hormones, genetic and epigenetic factors, cytokines, chemokines, and components of the extracellular matrix have been linked with the occurrence of fibroids [4]. More recent studies suggest that not only oestrogen but, in particular, progesterone plays an important role in the occurrence and growth of fibroids.

According to Kim and Sefton [12], not only does the mode of action of progesterone involve classic nuclear receptor effects on gene regulation, but there are also indications that the progesterone receptors (PR) directly activate signal pathways, interact with growth factor signal systems, and hence promote the proliferation and viability of fibroids.

### 1.5. Therapeutic Options

Due to the clinical symptoms, such as severe bleeding, pain in the pelvic region, and infertility, treatment is required in approximately a third of patients with uterine fibroids [4]. According to Stewart et al. [5], only few randomised controlled studies have been performed so far, which compare the effectiveness of various treatment techniques with one another and take variables such as age, ethnicity, and the characteristics of the tumour into consideration at the same time. In addition to various surgical techniques (e.g., hysterectomy or organ-preserving minimally invasive procedures) and radiological-gynaecological therapeutic techniques (e.g., uterine artery embolisation (UAE) and magnetic resonance imaging-guided focused ultrasound (MRgFUS)), medication-based treatment strategies are also possible therapeutic options. The current therapeutic options, including their advantages and disadvantages, have been described in detail in the publication by Rabe et al. [2].

Criteria which should be considered in the selection of the most suitable treatment are the patient's chronological age, the patient's wish to retain her organ and/or fertility, as well as tumour-associated factors such as the number, size, and location of the fibroids [4].

Many therapeutic options for fibroid patients involve surgical interventions; approximately a third to half of all hysterectomies are due to fibroids [5].

Consequently, there is a great need to develop new and effective therapeutic alternatives for women, in which organ retention and the retention of fertility have the priority. In these cases, a medication-based treatment is suitable. GnRH agonists, which trigger a temporary menopause with amenorrhoea by means of a reduction in the progesterone and oestrogen levels and therefore lead to an increase in the haemoglobin level, are increasingly more seldom employed therapeutically; their administration also leads to a reduction of the size of the fibroids. GnRH agonists are used preoperatively. Because of their side-effect profile, with climacteric complaints and reduced bone density, however, they can only be used over a brief period [4, 13]. As well, the targeted reduction of the uterine or fibroid volume is relatively quickly reversible after discontinuing the medication [13]. Another class of substances is the selective progesterone receptor modulators (SPRMs). One representative of this class is ulipristal acetate (UPA), which was approved in 2012 for the preoperative treatment of moderate to severe symptoms due to uterine fibroids in adult women of child-bearing age. Ulipristal acetate was also approved in May 2015 for intermittent treatment and it therefore presents a fully fledged alternative to a surgical procedure.

## 2. Selective Progesterone Receptor Modulators (SPRMs)

**Figure 2:**
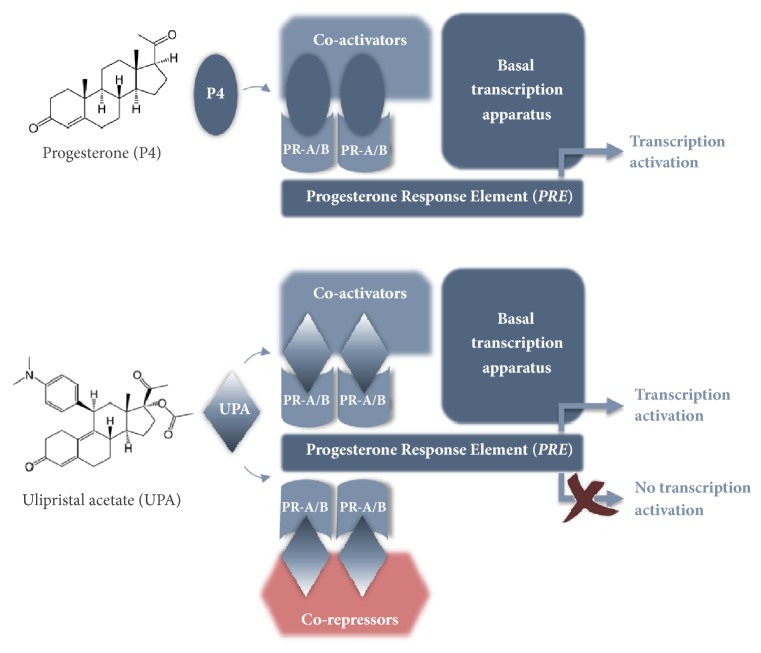
Mode of action of SPRMs according to Bouchard et al. [8]: SPRMs interact with coactivators and corepressors. In this way, the gene transcription is either inhibited or activated. This means that the stimulating or inhibiting effect of an SPRM is dependent on its chemical structure.

SPRMs are a substance class of synthetic steroids which have agonistic and/or antagonistic effects on the progesterone receptors (PR). Because of their structural similarity to progesterone, they are able to be taken up by its receptors and, depending on the change in the conformation of the receptor resulting from the bond, corepressors or coactivators are accumulated in the corresponding binding domain. Whether an SPRM has a more agonistic or antagonistic effect depends on its structure and the change in the conformation of the progesterone receptor and on the availability of coregulators (ratio of coactivators to corepressors) in a particular cell type (Figure 2). The activity of an SPRM is also affected by the tissue type and cell type, as well as the physiological context (e.g., pregnancy) [8, 14].

**Figure 3:**
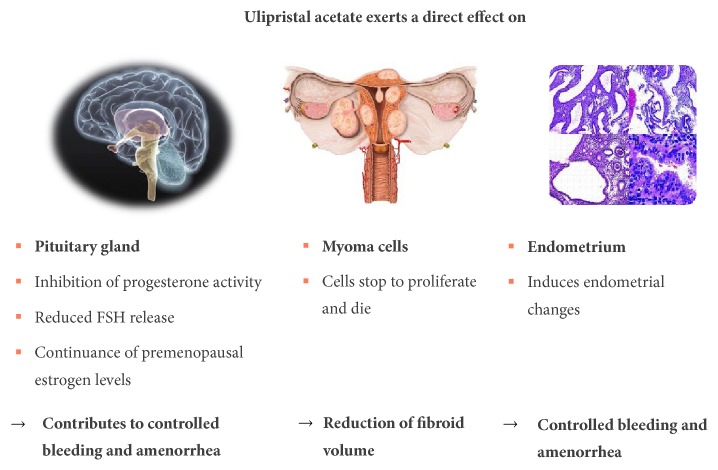
Effects of ulipristal acetate on the pituitary gland, endometrium, and fibroid according to Donnez and Dolmans [4].

Unlike GnRH agonists, which only affect the pituitary gland, where they cause a downregulation and a desensitisation of the GnRH receptors with a consecutive decrease in serum estradiol and progesterone levels, SPRMs have direct effects on the pituitary gland, the fibroid, and the endometrium [4] (Figure 3). Amenorrhoea is induced by means of the direct effect on the pituitary gland, by inhibiting ovulation (in approximately 80% of patients) and simultaneously maintaining the estradiol level in the mid-follicular range [15]. The direct effect on the endometrium is expressed as a suspension of uterine bleeding and in benign reversible changes to the endometrium (PAEC:* Progesterone Receptor Modulator Associated Endometrial Changes*) and in reversible endometrial thickening. In addition, SPRMs produce a reduction in the fibroids by inhibiting the cell proliferation and by inducing apoptosis [4].

## 3. Ulipristal Acetate (UPA)

ESMYA® (ulipristal acetate, 5 mg) is used to treat moderate to severe symptoms of uterine fibroids.

EMA's Pharmacovigilance Risk Assessment Committee (PRAC) has reviewed the benefits and risks with ESMYA®, following of serious liver injury, including liver failure leading to transplantation. The review of ESMYA® was initiated at the request of European Commission on 30 November 2017, under Article 20 of Regulation (EC) No 726/2004. The review was being carried out by the PRAC, the Committee responsible for the evaluation of safety issues for human medicines, which made a set of recommendations. On 8 February 2018, while the review was ongoing, the PRAC issued temporary measures to protect patients' health. On 19 February 2018 a so-called “Dear Doctor Letter” (“Rote-Hand-Brief”) [16] has been sent out to all gynaecologists, hepatologists, general practitioners, and pharmacies in Germany.

EMA's PRAC has completed its review of ESMYA® in May 2018. After considering all the evidence, the PRAC concluded that the medicine must not be used in women with liver problems and that certain other patients may start new treatment courses provided they have regular liver tests.

The PRAC has concluded that ESMYA® may have contributed to the development of some cases of serious liver injury. In 8 cases of serious liver injury, a role of ESMYA® in contributing to these cases is possible. It is estimated that around 765,000 patients have been treated with ESMYA® to date. The Committee has therefore made the following recommendations to minimize this risk:

- **ESMYA**®** must not be used in women with known liver problems.**
- **A liver function test should be performed before starting each treatment course** and treatment must not be started if liver enzyme levels are more than 2 times the upper limit of normal.
- **Liver function tests should be performed once a month during the first two treatment courses and two to four weeks after stopping treatment.** If the test is abnormal (liver enzyme levels more than 3 times the upper limit of normal), the doctor should stop treatment and closely monitor the patient.
- ESMYA® should be used for more than one treatment course only in women who are not eligible for surgery. Women who are about to have surgery should continue to use only one course.
- A card will be included in the box of the medicine to inform patients about the need for liver monitoring and to contact their doctor should they develop symptoms of liver injury (such as tiredness, yellowing of the skin, darkening of the urine, nausea, and vomiting).

On February 2018, while the review was ongoing, the PRAC had issued temporary recommendations that no new patients should be started on ESMYA®. Having finalized its review, the Committee has now concluded that new patients can start treatment in line with the above recommendations to minimize the risk of liver injury. The PRAC's recommendations will now be forwarded to the Committee for Medicinal Products for Human Use (CHMP) for the adoption of EMA's final opinion, and this will then go to the European Commission for a final legal decision. A letter will be sent to doctors to inform them of the new restrictions of use, which will become applicable after a Commission decision is issued. More information on the review procedure and recommendations can be found at the homepage of the EMA [17].

The clinical efficacy and safety of ulipristal acetate (UPA), both with regard to preoperative administration and with regard to an intermittent administration as long-term treatment for patients with symptomatic uterine fibroids, have been shown in several clinical studies (PEARL I–IV) as we will discuss in the following paragraphs. In patients suffering from heavy menstrual bleeding associated with uterine fibroids, repeated 3-month treatment courses with ulipristal acetate provide a medical alternative to surgery and have the potential to reduce the need for surgical intervention. No signal of hepatic toxicity was identified during the nonclinical or clinical trials of ESMYA®.

The following effects of the SPRM ulipristal acetate (UPA) have been described:

- **Fibroids: **the inhibition of proliferation, induction of apoptosis, and reduction of the extracellular matrix, probably due to an increased expression of metalloproteinase- (MMP-) 2, result in a reduction of the fibroids [18].
- **Pituitary gland: **an inhibition or delay of the ovulation with partially reduced LH, FSH, and estradiol levels within the physiological range (60 – 150 pg/ml, mid-follicular range) result in bleeding control without the appearance of the symptoms of an oestrogen deficiency [19].
- **Endometrium: **in most women, the interaction with endometrial progesterone receptors results in amenorrhoea and hence bleeding control [19, 20]. Under treatment with an SPRM, a thickening of the endometrium, which can be detected by ultrasound, may appear. This, however, does not represent an endometrial hyperplasia. This effect is asymptomatic and is reversible once the treatment has ended and menstruation has recommenced. In most cases, this endometrial thickening is caused by substance class-specific changes to the endometrium, which are referred to as PAEC (PAEC:* Progesterone Receptor Modulator Associated Endometrial Changes*) [14]. PAEC represent a distinct histological entity, which is viewed as benign and completely reversible [21, 22]. An inactive, weakly proliferating epithelium with asymmetry of the stromal and epithelial growth, which results in prominent cystically enlarged endometrial glands, which simultaneously exhibit the epithelial effects of oestrogen (mitotic) and gestagen (secretory), is characteristic of PAEC. Further characteristics are an apparently inactive and mitotically less active (weakly proliferating) endometrium, increased apoptosis in the glandular epithelium, and a compact stroma [14].

### 3.1. Clinical Evidence of Effectiveness

#### 3.1.1. Preoperative Application

UPA was approved for the preoperative treatment of moderate to severe symptoms due to uterine fibroids in adult women of child-bearing age in February 2012. The evidence of effectiveness for this indication is based on the PEARL I and PEARL II studies [11, 23]. The results of these studies are shown in Table 1. The objective of the PEARL I study was to examine the effectiveness and tolerance of UPA in doses of 5 mg per day and 10 mg per day in comparison with a placebo in women with symptomatic uterine fibroids prior to scheduled surgery. All patients were given 80 mg of depot iron(II)-sulphate per day during the treatment. PEARL II is a noninferiority trial for the verification of the effectiveness of UPA versus the GnRH agonist, leuprolide acetate, in terms of the reduction of increased uterine bleeding in fibroid patients, for whom surgery had been scheduled.

In these studies, it was evident that bleeding due to fibroids was attenuated or ceased in over 90% of the patients thanks to the administration of UPA. The amenorrhoea rate under treatment with 5 mg and 10 mg of UPA was between 73% and 89%. In PEARL I, correction of the anaemia (Hb > 12 g/dl) was achieved more frequently under UPA than under the placebo (85 – 89% versus 77%) [14, 23].

Bleeding control was more rapidly achieved under UPA than with the GnRH agonist. Under UPA, amenorrhoea appeared after 5–7 days (median) and only after 21 days under leuprolide acetate [11]. According to Donnez et al. [9], 80% of the patients in both studies showed a clinically relevant reduction of > 25% of the volume of the fibroids; in 50% of the women, the fibroid volume decreased by 50%. In both UPA treatment groups, the estradiol level was in the mid-follicular range, while, for the patients treated with leuprolide acetate, this fell to postmenopausal levels [14]. Hot flushes appeared significantly more frequently under GnRH treatment. In one group of the women who did not undergo surgery following the completion of the study, it was observed that the fibroid reduction achieved under UPA persisted for up to 6 months after the completion of the study, while, in the women treated with the GnRH agonist, fibroid growth restarted quickly [11, 14]. A further study, PEARL III, was initiated on the basis of these results in order to investigate the effectiveness and safety of intermittent long-term treatment with 10 mg of UPA per day, each for 12 weeks, for the treatment of symptomatic uterine fibroids.

#### 3.1.2. Repeated Intermittent Treatment

PEARL III [24] was an open study, in which a double-blind 10-day administration of norethisterone acetate (NETA) or a placebo followed each 12-week treatment interval with UPA (daily dose: 10 mg). Up to a total of four treatment intervals with UPA were possible. The objective of this multicentre clinical phase III study was to investigate the clinical effectiveness and safety of intermittent long-term treatment with UPA. The results of this study show that the repeated application of UPA can maximise its potential benefit in terms of bleeding control and fibroid reduction [9, 14]. In less than 10% of patients, transient endometrial thickening appeared following each treatment interval. Hyperplasias or adenocarcinomata of the endometrium were not observed at any point in time [24]. The administration of NETA after each UPA treatment interval had no effect on the size of the fibroids or on the histology of the endometrium. The most important results of PEARL III are outlined in Table 1. At this point, a detailed account of the results is dispensed with since the 10 mg per day dose for the treatment of fibroids is not approved in Germany.

In the latest PEARL IV study, the effectiveness and safety of UPA were investigated in 4 consecutive 12-week treatment intervals [10, 25]. A 3-month therapy-free follow-up was performed after each treatment interval. PEARL IV was a double-blind randomised study, in which the patients were given treatment with UPA in a daily dose of 5 mg or 10 mg. The treatment commenced within the first four days of menstruation, the next treatment interval started only with the commencement of the 2nd bleeding after the conclusion of the previous treatment interval. In total, 451 fibroid patients with severe bleeding were included in the study, of whom 228 were given a daily dose of 5 mg and 223 were treated with 10 mg of UPA. The primary effectiveness criterion was the percentage of the patients with amenorrhoea (definition: ≤ 1 day with spotting in a 35-day interval) following all four treatment intervals. The rate of amenorrhoea and the period until the appearance of amenorrhoea after each individual treatment interval, bleeding control (definition: no severe bleeding and bleeding for a maximum of 8 days during the last 56 days of a treatment interval) after each treatment interval, the fibroid volume, pain, and the quality of life were secondary effectiveness criteria. The rate of premature study dropouts on the grounds of safety (i.e., clinically significant changes in the gynaecological breast examination, ovarian ultrasound, ECG, laboratory diagnostics, vital signs, endometrial thickness, and histology, in accordance with the study protocol) and the frequency of side-effects were defined as endpoints for safety.

**Figure 4:**
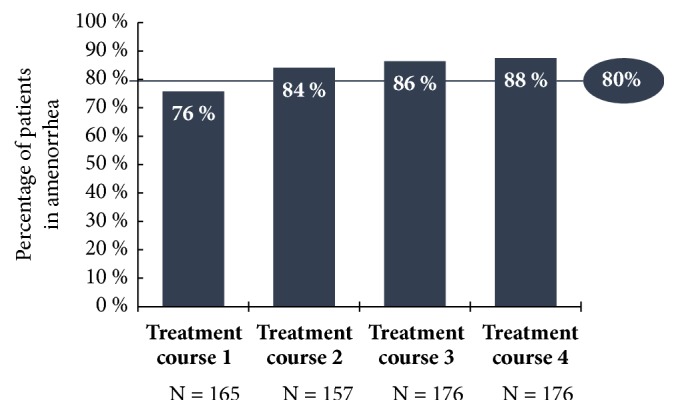
Percentage of patients with amenorrhoea at the end of each treatment interval with 5 mg of UPA (PP4; PEARL IV).

**Figure 5:**
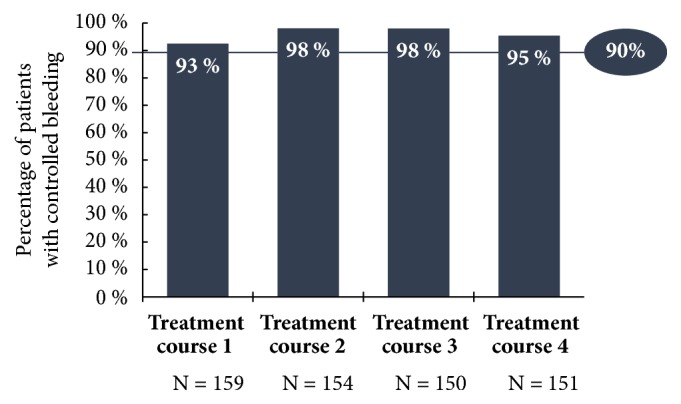
Percentage of patients with bleeding control at the end of each treatment interval with 5 mg of UPA (PP4; PEARL IV).

**Figure 6:**
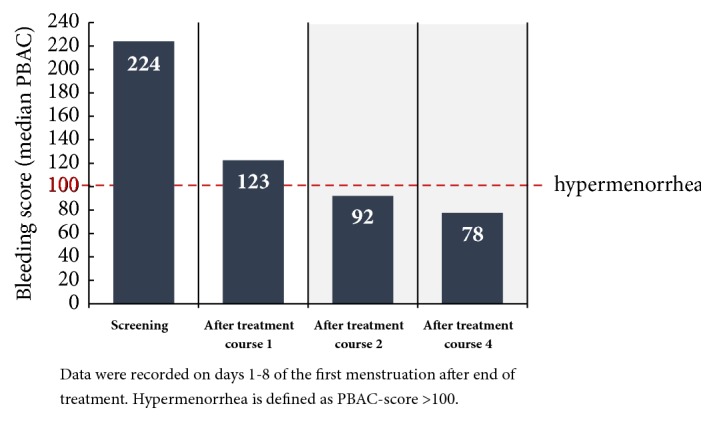
Bleeding intensity (PBAC median) of the first menstruation following the completion of a treatment interval (FAS1; PEARL IV).

**Figure 7:**
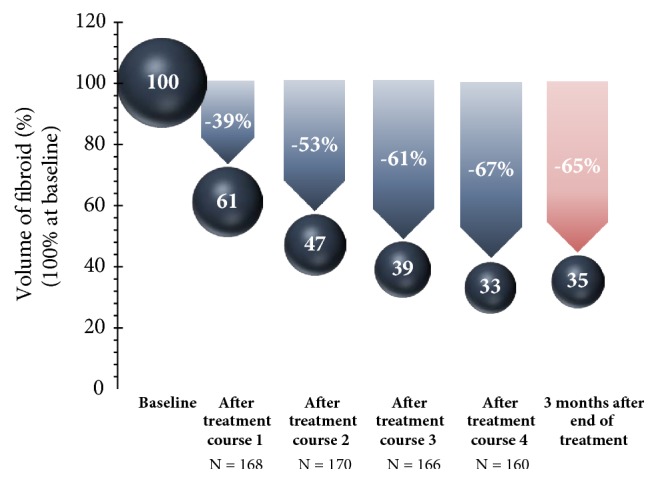
Volume change in the 3 largest fibroids (median) in comparison with the initial value.

**Figure 8:**
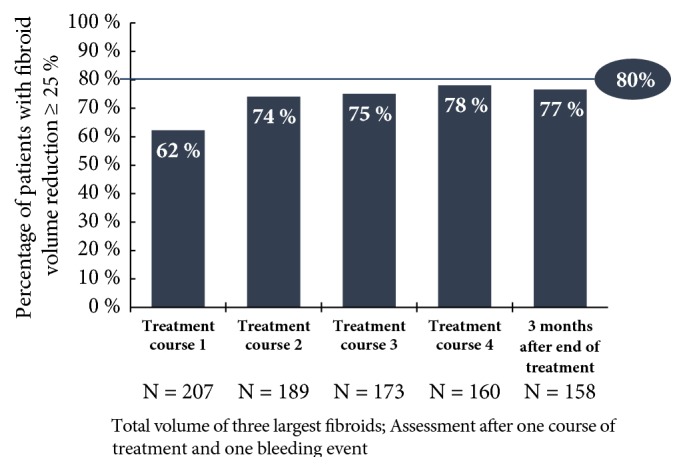
Percentage of patients with clinically significant reduction of the volume of the fibroids of ≥ 25% (FAS1) [9].

Only the results for the 5 mg UPA dose are represented in this study since only this has been approved in Germany for the treatment of fibroids. Unless otherwise stated, all data given here originate in the publication by Donnez et al., including the supplementary evaluation in the attachment (2016; Part II), and refer either to the Full Analysis Set (FAS1: all patients who were given the study medication at least once in the 1st treatment interval) or to the Per Protocol Set (PP4: all patients who were given the study medication over at least 56 days in the 4th treatment interval). In respect of the primary effectiveness criterion, an amenorrhoea rate of 48.7% was determined across all four treatment intervals. In the individual treatment intervals, the amenorrhoea rates in the Per Protocol Set (PP4) were 75.8%, 84.1%, 86.4%, and 87.5% (Figure 4). The corresponding results of the FAS1 over the individual treatment intervals were consistently high (71.8%, 74.1%, and 73.3% after intervals 1, 2, and 3) and amenorrhoea occurred in approximately 70% of the women after the 4th UPA treatment interval. The period until the occurrence of amenorrhoea in all treatment intervals was 5-6 days (median). The effects of 5 mg of UPA in respect of the bleeding control in the course of the study are represented for the Per Protocol Set (PP4) in Figure 5. In the Full Analysis Set 1, the bleeding was under control in 73.3% of the patients at the end of the 4th treatment interval; at the same time, the PBAC (pictorial bleeding assessment chart) had decreased from 224 to 77.5 (median) (Figure 6). In regard to the bleeding intensity during the treatment breaks, it was observed that a bleeding intensity PBAC of < 100 is achieved with at least two 12-week treatment intervals under UPA, which is therefore below the threshold value for hypermenorrhoea (PBAC = 100). The fibroid volume (the volume of the 3 largest fibroids was measured) decreased continually with each treatment interval in comparison with the initial value (Figure 7). The percentage of patients with a clinically significant reduction of the fibroid volume (≥ 25%) increased from 62.3% after the 1st treatment interval to 78.1% after the 4th treatment interval. At 76.6% (Figure 8), this value was largely unchanged in the follow-up examination 3 months after the end of the treatment. The greatest decrease in the fibroid volume was observed after the first two treatment intervals, which shows that a second treatment interval substantially increases the therapeutic effect [9].

**Figure 9:**
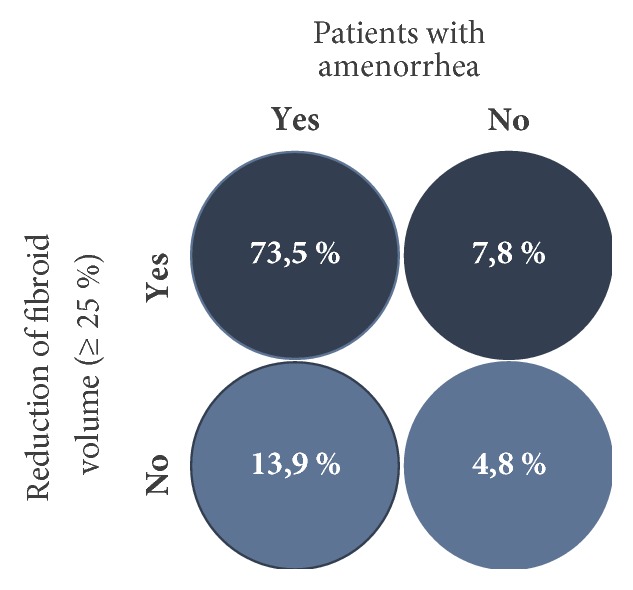
Percentage of patients with amenorrhoea and clinically significant reduction of the volume of the fibroids of ≥ 25% [10].

After the 4th treatment interval, 73.5% of all women had amenorrhoea and a decrease in the fibroid volume of ≥ 25% [24]. Only 4.8% of the women treated with UPA had neither amenorrhoea nor a reduction of the fibroid volume of ≥ 25% (Figure 9). Overall, 95% of the patients benefited from the long-term interval treatment with 5 mg of ulipristal acetate, so that no invasive procedure was required during the entire period of observation in over 95% of the cases.

**Figure 10:**
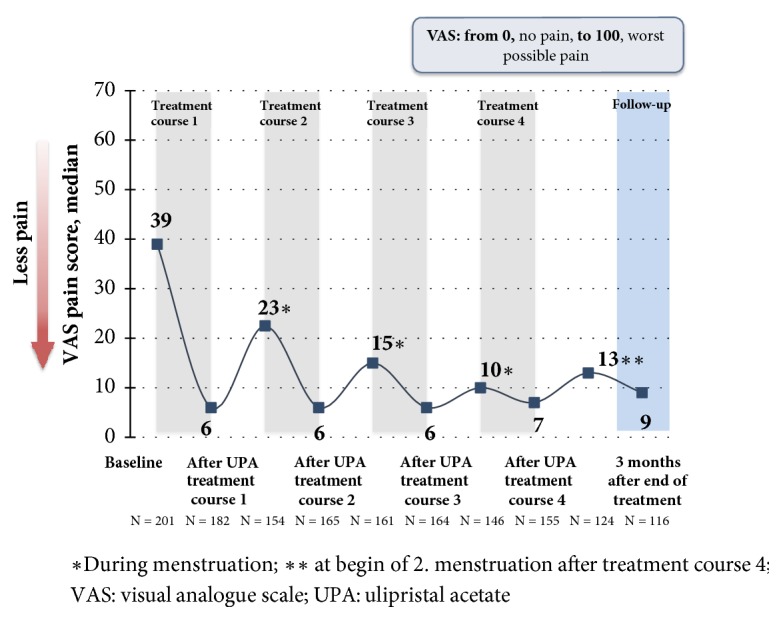
Effect of 5 mg of UPA on the course of pain (VAS). Outline of the median values (FAS1) [11].

**Figure 11:**
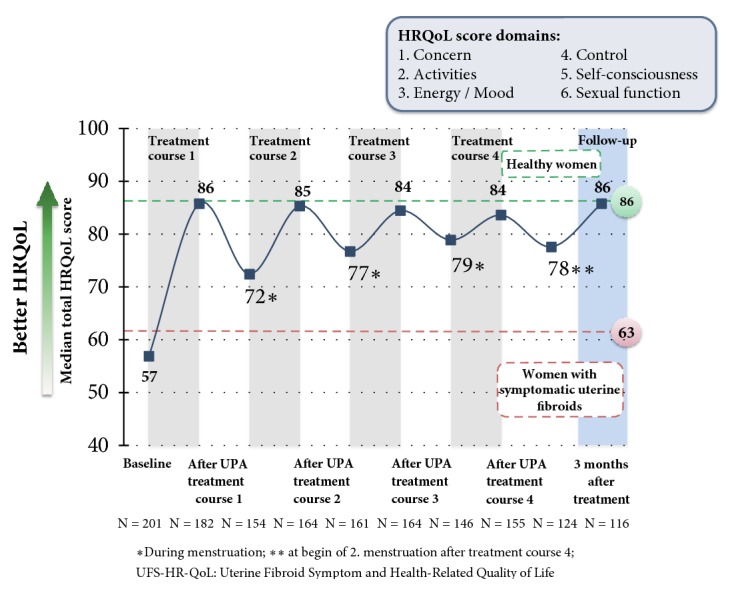
Effect of 5 mg of UPA on the quality of life (HR QoL). Outline of the median values (FAS1).

Pain, which was recorded by means of visual analogue scale (VAS: 1 – 100), improved under the treatment and decreased from the initial score of 39 (median) after the 4th treatment interval to a value of 7 (median). The pain also remained at a low level during the treatment breaks and at the end of the follow-up and ranged between 9.0 and 22.5 [9] (Figure 10). The quality of life, which was assessed by means of the UFS QoL (Uterine Fibroid Symptom and Quality of Life) questionnaire, decreased from an initial value of 50 (median) to 15.6 after the 4th treatment interval, where this value is consistent with healthy women [9, 10]. The results of the HR QoL (Health Related Quality of Life) in the course of the study are shown in Figure 11.

The estradiol levels were in the mid-follicular range over the entire duration of the study. The Hb value showed an increase, from the initial value of 12.55 g/dl to 13.10 g/dl, after the 4th treatment interval, although, unlike the Pearl I study, anaemia was not an inclusion criterion in this study.

**Figure 12:**
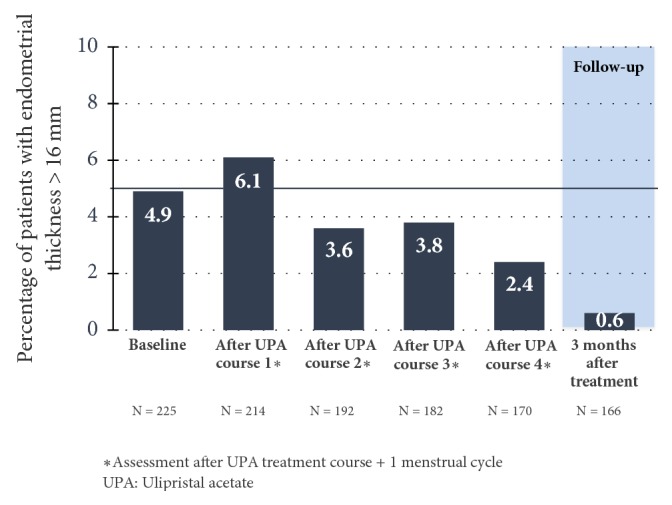
Percentage of patients with an endometrial thickness of > 16 mm (Safety Population) [10].

Undesirable reactions, which the doctor ascribed to UPA as causal, occurred in 20.4%, 13.0%, 4.7%, and 6.1% of the patients (treatment intervals 1, 2, 3, and 4). With an increasing number of treatment intervals, side-effects decreased steadily. The most frequently observed side-effects were headaches and hot flushes. After a temporary increase in the endometrial thickness in the 1st treatment interval, an endometrial thickness of > 16 mm was less and less frequently measured in the following treatment intervals [9, 10] (Figure 12). Over the entire study, according to the biopsy and histology examination, 6 cases of hyperplasia occurred. These reduced as the treatment progressed and/or during the follow-up period [9].

**Figure 13:**
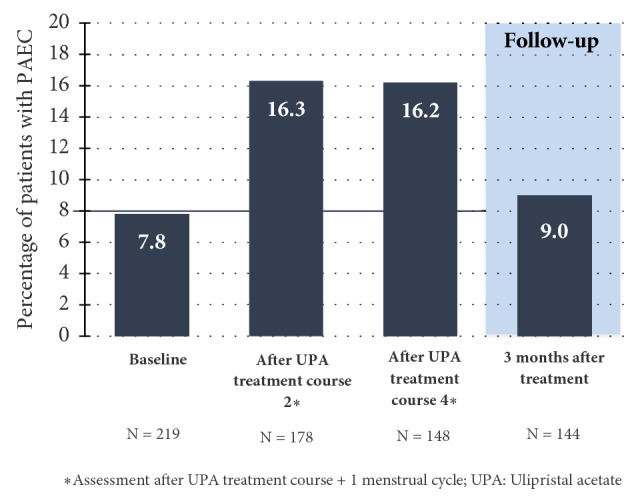
Percentage of patients with nonphysiological changes of the endometrium (PAEC) (Safety Population).

The frequency of nonphysiological endometrial changes (PAEC) at the commencement of the study was 7.8% and increased to 16.3% and 16.2% until the end of the 2nd and 4th treatment interval. In the follow-up after 3 months, nonphysiological changes of the endometrium were observed in 9% of the patients, which broadly corresponds with the initial values prior to the commencement of the study [10]. Hence, long-term intermittent treatment with 5 mg of UPA does not result in an increased incidence of PAEC (Figure 13). The further findings in the physical examination, vital signs, ECG, and sonography of the ovaries produced no reservations in regard to the safety of UPA.

The results of PEARL IV demonstrate the effectiveness and safety of UPA as an intermittent treatment for symptomatic fibroid patients, and so the approval of UPA with a daily dose of 5 mg was extended to long-term interval therapy (LIT), not limited in terms of time, in May 2015.

## 4. Significance of UPA in Clinical Practice

The application of UPA for intermittent long-term treatment for fibroids is evaluated in current publications as an asset since; for most patients, this results in rapid and reliable bleeding control, a reduction in the size of the fibroids and an improvement in the symptoms and, in many cases, can prevent a hysterectomy or fibroid enucleation [26]. Donnez et al. also share this view [9]. They describe a legitimate status for UPA in the medication-based treatment of fibroids in regard to avoiding a surgical intervention or at least as a preoperative supplementary measure.

In their latest paper, Donnez and Dolmans [4] present the various possible applications of UPA in the treatment of fibroids, taking the age of the patient, the severity of the symptoms, the wish of the patient in regard to the retention of the organ and fertility, and the location and the size of the fibroids into consideration.

*Type 1 Fibroids*. They are < 3 cm in size: hysteroscopic myomectomy.

When the size of the fibroid is > 3 cm or with anaemia, medication-based treatment with one to two treatment intervals with UPA is conducted, each over 12 weeks. When there is a good response to the treatment, a surgical procedure may possibly be avoided.

*Type 2 Fibroids or Multiple Type 2*-*5 Fibroids*. Long-term intermittent treatment with two 12-week UPA treatment intervals may be indicated for women of child-bearing age with a desire to have children. In the case of a good response (fibroid reduction of ≥ 25 – 50%, no deformation of the uterine cavity) or very good (fibroid reduction of > 50%, no deformation of the uterine cavity) response to the treatment, the possibility of a normal conception is a given. When there is an inadequate fibroid volume and/or bleeding response and when there is existing deformation of the uterine cavity, a hysteroscopic myomectomy may be necessary.

For women of child-bearing age who do not wish to have children or for premenopausal women who wish to retain the organ, long-term intermittent treatment with four 12-week UPA treatment intervals can be recommended. When there is a good response (volume reduction of ≥ 25%, bleeding control), the treatment can be suspended until the recurrence of symptoms; otherwise, a surgical procedure is indicated. For women of child-bearing age, a fibroidectomy can be performed when there is an inadequate response to the medication-based treatment. A UAE is also a possibility for premenopausal women who wish to retain the organ.

## 5. Conclusion

UPA is suitable for the treatment of fibroids, both for preoperative use and for intermittent long-term treatment. It has been shown in clinical studies that bleeding is successfully controlled by the administration of 5 mg per day, over 12-week treatment intervals each time. For most women, amenorrhoea starts within a few days and anaemia is corrected. UPA also causes a reduction in the size of the fibroids, an improvement in the pain, and, overall, results in a distinct improvement in the quality of life of the affected patients. In terms of bleeding control and fibroid reduction, the effects are increased without an increased rate of side-effects or the occurrence of endometrial changes by the initial administration of a least two UPA treatment cycles. Clinical practice experience shows that, after a twofold treatment (each of three months) in particular, the interval before a required third treatment can often be extended further (between 6 and 12 months) [27].

Despite the PRAC's new measures to minimize the risk of rare but serious liver injury the use of the SPRM ulipristal acetate still presents new therapeutic perspectives, particularly for symptomatic fibroid patients for whom the wish to retain the organ or fertility is a priority.

## Figures and Tables

**Table 1:** Most important results from studies PEARL I–III [11, 23, 24].

	*Study design*	*Study drug/- duration*	*Inclusion criteria*	*Study population*	*Primary endpoints and results*	*Secondary endpoints and results*
*PEARL I [23]*	*double-blind, randomized,* *placebo controlled, multicenter, parallel group*	*UPA 5 mg/d* *vs.* *UPA 10 mg/d* *vs.* *placebo* *Duration:* *12 Weeks* *All patients* *received iron* *supplementation*	*women with uterine fibroids,hypermenorrhea* *(PBAC> 100), anemia (Hb < 10,2 g/dl),* *eligible to undergo fibroid surgery after the end of the treatment period*	*UPA 5 mg:* N = 96 *UPA 10 mg:* N = 98 *Placebo:* N = 48	*Percendays of patients with control of uterine bleeding (PBAC* *< 75) at week 13:* *UPA 5 mg: 91 *%*∗* *UPA 10 mg: 92 *%*∗* *Placebo: 19 *% *∗P < 0,001* *Reduction of fibroid volume compared to baseline* *(median) at week 13:* *UPA 5 mg: -21,2 *%*∗* *UPA 10 mg: -12,3 *%∗∗ *Placebo: +3 *% *∗P = 0,002* *∗∗P = 0,006*	*Percendays of patients in amenorrhea at week 13:* UPA 5 mg: 73 %*∗* UPA 10 mg: 82 %*∗* Placebo: 6 % *∗P *< 0,001 *Menstrual bleeding:* PBAC at baseline (median) UPA 5 mg: 386 UPA 10 mg: 330 Placebo: 376 vs. at week 13: UPA 5 mg: -329*∗* UPA 10 mg: -326*∗* Placebo: -59 *∗P *< 0,001

*PEARL II [11]*	*double-blind, randomized* * parallel group,* *double-dummy, noninferiority trial*	*UPA 5 mg/d* *vs.* *UPA 10 mg/d* *vs.* *Leuprorelin* *Acetate* *(LA) 3,75 mg i.m.* */1x Month* *Duration: 12 weeks*	*Women with uterine* *fibroids,* *Hypermenorrhea,* *all patients eligible for* *uterine fibroid surgery*	*UPA 5 mg:* N = 93 *UPA 10 mg:* N = 95 *LA:* N = 93	*Percendays of patients with* *controlled bleeding (PBAC < 75)* *at week 13:* *UPA 5 mg: 90 *%*∗* *UPA 10 mg: 98 *%∗∗ *LA: 89 *% *∗1,2 (95 *%* CI: -9,3 to 11,8)* *∗∗8,8 (95 *%* CI: 0,4 to 18,3)*	*Percendays of patients with* *amenorrhea at week 13:* UPA 5 mg: 75 % UPA 10 mg: 89 % LA: 80 % *Median change in fibroid* *volume of the 3 largest* *fibroids at baseline and at week 13*: UPA 5 mg: -36 % UPA 10 mg: -42 % LA: -53 %

*PEARL III [24]*	*Long-term phase III* *UPA treatment* *Courses* *Open* *NETA treatment corses:* *double-blind, placebo controlled, randomized*	*Per treatment* *course:* *UPA 10 mg/d* *for 12 weeks* *followed by* *NETA 10 mg/d* *for 10 days or* *placebo* *for a total of 4 treatment courses*	*Women with* *symptomatic uterine* *fibroids, eligible for* *uterine fibroid surgery*	*UPA treatment* *Treatment course 1* N = 132 *Treatment course 2* N = 131 *Treatment course 3* N = 119 *Treatment course 4* N = 107	*Patients in amenorrhea after each* *treatment course* *Treatment course 1: *79,5 % *Treatment course 2: *88,5 % *Treatment course 3: *88,2 % *Treatment course 4: 8*9,7 %	*Median change in fibroid* *volume of the 3 largest* *fibroids at baseline* *Treatment course 1:*-45 % *Treatment course 2: -63 *% *Treatment course 3:*-67 % *Treatment course 4: -72 *% *time to amenorrhea* *Treatment course 1: *4 Days *Treatment course 2: *2 Days *Treatment course 3: *3 Days *Treatment course 4: 3 *Days

PBAC: pictorial blood-loss assessment chart; UPA: ulipristal acetate; LA: leuprorelin acetate; NETA: norethisterone acetate.
