# Corrigendum to “Selective Progesterone Receptor Modulators for the Medical Treatment of Uterine Fibroids with a Focus on Ulipristal Acetate”

**DOI:** 10.1155/2018/6124628

**Published:** 2018-11-28

**Authors:** Thomas Rabe, Nicole Saenger, Andreas D. Ebert, Thomas Roemer, Hans-Rudolf Tinneberg, Rudy Leon De Wilde, Markus Wallwiener

**Affiliations:** ^1^Deutsche Gesellschaft für Gynäkologische Endokrinologie und Fortpflanzungsmedizin, Germany; ^2^Klinikum für Frauenheilkunde und Geburtshilfe, Frankfurt/M, Germany; ^3^Praxis für Frauengesundheit, Gynäkologie und Geburtshilfe, Berlin, Germany; ^4^Evangelisches Krankenhaus Weyertal gGmbH, Köln, Germany; ^5^Universitätsklinik Gießen und Marburg, Campus Gießen, Germany; ^6^Klinik für Frauenheilkunde, Geburtshilfe und Gynäkologische Onkologie; Universitätsklinik für Gynäkologie, Pius-Hospital Oldenburg, Germany; ^7^Universitäts-Frauenklinik Heidelberg, Germany

In the article titled “Selective Progesterone Receptor Modulators for the Medical Treatment of Uterine Fibroids with a Focus on Ulipristal Acetate” [1], the affiliation of the third author was incorrect. The corrected affiliation is shown above. In addition, the Conflicts of Interest section should be updated as follows:

The authors have each previously received financial support from Gedeon Richter, who market ulipristal acetate as ESMYA®. Thomas Rabe received fees for lectures, publications, and participation on the advisory board before retiring in 2016, Thomas Roemer received fees for lectures and consultancy work, Nicole Sänger has been a consultant for Gedeon Richter and has received travel expenses and lecture fees, Hans-Rudolf Tinneberg received fees for lectures, Andreas D. Ebert received financial support for lectures and the establishment of the Myom-Netwerk, Markus Wallwiener received a honorarium for a congress presentation, and Rudy Leon De Wilde was reimbursed for costs concerning a presentation on myoma surgery.
